# Supplementary material for: Optoelectronic Enhancement of Perovskite Solar Cells through the Incorporation of Plasmonic Particles
Source: Micromachines (Basel). 2022 Jun 25;13(7):999. doi: 10.3390/mi13070999 (PMC9323966; doi:10.3390/mi13070999)
Supplement: Supplementary file 1 [file micromachines-13-00999-s001.zip › micromachines-1780286-supplementary.pdf]

Optoelectronic Enhancement of Perovskite Solar Cells through the Incorporation of Plasmonic Particles

Mohamed Salleh Mohamed Saheed [1,2], Norani Muti Mohamed [1,2], Balbir Singh Mahinder Singh [1,2], Mohamed Shuaib Mohamed Saheed [1,3,*] and Rajan Jose [4,5,*]

[1] Centre of Innovative Nanostructures and Nanodevices (COINN), Universiti Teknologi PETRONAS,
Seri Iskandar 32610, Malaysia; mohamed.salleh87@gmail.com (M.S.M.S.); noranimuti_mohamed@utp.edu.my (N.M.M.); balbir@utp.edu.my (B.S.M.S.)
[2] Department of Fundamental and Applied Sciences, Universiti Teknologi PETRONAS, Seri Iskandar 32610, Malaysia
[3] Department of Mechanical Engineering, Universiti Teknologi PETRONAS, Seri Iskandar 32610, Malaysia
[4] Center for Intelligent Materials, Universiti Malaysia Pahang, Kuantan 26300, Malaysia
[5] Faculty of Industrial Sciences & Technology, Universiti Malaysia Pahang, Kuantan 26300, Malaysia
* Correspondence: shuaib.saheed@utp.edu.my (M.S.M.S.); rjose@ump.edu.my (J.R.)

Supporting Information

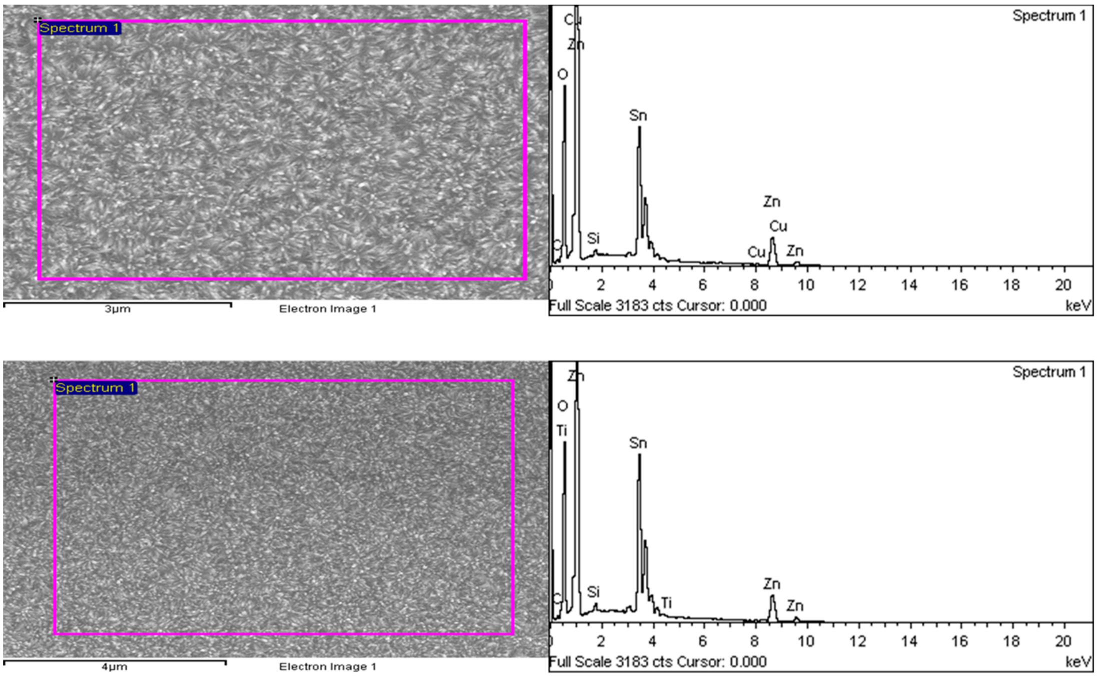

Figure S1: EDX spectrum of ZnO NFs deposited with a) Ag, b) Cu and c) Ti.
